# Improved Hybrid Genome Assemblies of Two Strains of *Bacteroides xylanisolvens*, SD_CC_1b and SD_CC_2a, Obtained Using Illumina and 454 Sequencing Technologies

**DOI:** 10.1128/genomeA.00237-14

**Published:** 2014-04-03

**Authors:** Thiruvarangan Ramaraj, Anitha Sundararajan, Faye D. Schilkey, Vito G. DelVecchio, Mildred Donlon, Cherie Ziemer, Joann Mudge

**Affiliations:** a National Center for Genome Resources (NCGR), Santa Fe, New Mexico, USA; b Vital Probes, Inc. (VPI), Mayfield, Pennsylvania, USA; c Defense Advanced Research Projects Agency (DARPA), Arlington, Virginia, USA; d National Laboratory for Agriculture and the Environment, United States Department of Agriculture, Agricultural Research Service (USDA-ARS), Ames, Iowa, USA

## Abstract

*Bacteroides xlyanisolvens* strains (SD_CC_1b, SD_CC_2a) isolated from human feces were grown on crystalline cellulose. Cellulolytic properties are not common in *Bacteroides* species. Here, we report improved genome sequences of both of the *B. xlyanisolvens* strains.

## GENOME ANNOUNCEMENT

*Bacteroides xlyanisolvens* is commonly found in the human gut (1). The type strain, XB1A, has high xylanase activity and extensively ferments xylan (2). We isolated two strains, SD_CC_1b and SD_CC_2a, using a substrate-depleted medium (3) with crystalline cellulose as the only carbohydrate, at the USDA-ARS National Laboratory for Agriculture and the Environment (Ames, IA). Analysis of the postfermentation cellulose structure indicated that SD_CC_1b and SD_CC_2a might have unique degradation properties (data not shown). In order to further determine the genomic basis for these results we sequenced the whole genome.

DNA was isolated using a DNeasy blood and tissue kit (Qiagen). PCR amplification of 16S rRNA genes was done with 27F (5′ AGAGGTTTGATCMTGGCTCAG 3′) and 1492R (5′ TACGGYTACCTTGTTACGACTT 3′) primers (Invitrogen) and a DYAD DNA Engine thermocycler (MJ Research) to determine phylogentic species placement. The PCR mixture (50 µl) contained 1× Qiagen PCR buffer, 1.25 U of *Taq* polymerase (Qiagen), 0.25 mM of each deoxynucleoside triphosphate (dNTP) (Amresco), 25 pmol of each primer, and 80 ng of template DNA. Amplified products were cleaned using QIAquick 96 PCR purification (Qiagen) and sequenced by the Iowa State University DNA Sequencing and Synthesis Facility (Ames, IA) using an ABI Prism 377 sequencer.

We built upon an assembly using a total of 337,702 reads (215.6 million bp and ~35-fold) for SD_CC_1b (GenBank accession no. SRX015718) and 245,608 reads (143 million bp and ~23-fold) for SD_CC_2a (SRX015722), generated using 454 GS-FLX and assembled using a Newbler assembler (4). A total of 6,059,812 bp in 236 contigs (*N*_50_, 60,820 bp) and 6,050,198 bp in 305 contigs (*N*_50_, 40,148 bp) were generated for SD_CC_1b (GenBank accession no. ASM17821v1) and SD_CC_2a (ASM17829v1), respectively. We added a small insert (~374 bp) library for each strain prepared using the standard Illumina (HiSeq2000) protocol. After screening for sequencing artifacts and ΦX contamination, 50.6 million (~1,687-fold) and 47.1 million (~1,569-fold) 100-bp paired-end reads were generated for SD_CC_1b and SD_CC_2a, respectively. The coverage calculated was based on a genome size estimate of ~6 Mbp. Scaffolds were generated from Illumina reads using the ABySS (v 1.3.4) assembler (5). Ilumina and 454 assemblies were merged using PHRAP (6), an overlap layout consensus (OLC) style assembler. The SD_CC_1b assembly had a total of 6,484,037 bp (60 scaffolds, with a GC of 42% and an *N*_50_ of 230,871 bp), and SD_CC_2a had a total of 6,228,594 bp (68 scaffolds, with a GC of 42% and an *N*_50_ of 214,376 bp). The assemblies were greatly improved, and the number of sequences decreased from 236 to 60 in SD_CC_1b and 305 to 68 in SD_CC_2a. The *N*_50_ increased from 60,820 bp to 230,871 bp and 40,148 bp to 214,376 bp for SD_CC_1b and SD_CC_2a, respectively. The quality levels of both assemblies were assessed by mapping Illumina reads back to the assembly using BWA (7). High percentages of uniquely aligning reads (~87% mapped back with ~86% properly paired and ~85% mapping uniquely for both strains) to the final genome in both strains validated the *de novo* assembly process. Gene prediction and annotation for both strains were performed using the RAST (8) server incorporating GLIMMER (9, 10). A total of 5,521 protein-coding and 83 RNA sequences were predicted for SD_CC_1b and 5,328 protein-coding and 75 RNA sequences were predicted for SD_CC_2a.

### Nucleotide sequence accession numbers.

Draft genome sequences for both strains have been deposited in the European Nucleotide Archive, under accession numbers CBXG000000000 (SD_CC_1b) and CBXH000000000 (SD_CC_2a).
